# Expression of Toll-like receptors 2 and 9 in cells of dog jejunum and colon naturally infected with *Leishmania infantum*

**DOI:** 10.1186/1471-2172-14-22

**Published:** 2013-05-14

**Authors:** Maria M Figueiredo, Izabela FG Amorim, Aldair JW Pinto, Vítor S Barbosa, Lucélia de Jesus Pinheiro, Beatriz Deoti, Ana MC Faria, Wagner L Tafuri

**Affiliations:** 1 Departamento de Patologia Geral, Faculdade de Medicina, Universidade Federal de Minas Gerais, Avenida Antônio Carlos, Pampulha, 6627, Belo Horizonte, Minas Gerais, Brazil; 2 Departamento de Cirurgia, Faculdade de Medicina, Universidade Federal de Minas Gerais, Avenida Professor Alfredo Balena, 190, Belo Horizonte, Minas Gerais, Brazil; 3 Departamento de Bioquímica e Imunologia, Instituto de Ciências Biológicas, Universidade Federal de Minas Gerais, Avenida Antônio Carlos, Pampulha, 6627, Belo Horizonte, Minas Gerais, Brazil

**Keywords:** Canine visceral leishmaniasis, Jejunum and colon, Toll-like receptors 2 and 9, Parasite burden, *Leishmania infantum*

## Abstract

**Background:**

Infection with parasite protozoa is a long-term health issue in tropical and subtropical regions throughout the world. The Toll-like receptor (TLR) signaling pathway is one of the first-responding defense systems against *Leishmania*. The aim of this study was to investigate the expression of TLR2 and TLR9 in jejunum and colon and its correlation with CD11c, CD11b, and CD14 receptors used as markers for dendritic cells and macrophages.

**Methods:**

Twenty four dogs infected with *Leishmania infantum* were used in this study. Cytometry was carried out in lamina propria cells from jejunum and colon using markers for TLR2, TLR9, CD11b, CD11c and CD14.

**Results:**

Cellular inflammatory exudate was diffuse in the mucosa and submucosa, predominately comprising mononuclear cells: plasma cells, macrophages, and lymphocytes. Despite the parasite load, microscopy showed no erosion was evident in the epithelial mucosa layers. The colon harbored more parasites than the jejunum. Flow cytometry revealed higher frequency of TLR2^+^ and CD11c^+^ dendritic cells in the colon than in the jejunum. Conversely, TLR9-expressing cells were more frequent in jejunum. Moreover, frequency of macrophages (CD11b^+^ and CD14^+^) expressing simultaneity TLR9 were lower in the colon than in jejunum, while CD11c^+^ cells predominated in the colon. Despite of the negative ELISA serum results, IL-10 and TNF-α were higher in jejunum than colon of infected animals. However, IL-4 was higher in colon than jejunum of infected animals. A higher expression these cytokines were demonstrated in infected dogs compared to uninfected dogs.

**Conclusions:**

There was no correlation between clinical signs and pathological changes and immunological and parasitological findings in the gastrointestinal tract in canine visceral leishmaniasis. However, jejunum showed a lower parasite load with increased frequency and expression of CD11b, TLR9, CD14/CD11b/TLR9 receptors and IL-10 and TNF-α cytokines. Conversely, the colon showed a higher parasite load along with increased frequency and expression of TLR2, CD11c receptors, and IL-4 cytokine. Thus, *Leishmania infantum* is able to interfere in jejunum increased expression of TLR2, TLR9, CD11b, CD14, CD14/CD11b/TLR9 receptors, IL-10, and TNF-α; and in colon increased expression of CD11c, TLR2, TLR9, CD11b, CD14 e, CD14/CD11b/TLR9 receptors, IL-10, and TNF-α.

## Background

Canine visceral leishmaniasis (CVL) is a worldwide zoonosis prevalent in approximately 50 countries in the Mediterranean basin, Middle East, and South America. In Brazil, the parasite *Leishmania infantum* is the cause of CVL, and the sandfly *Lutzomyia longipalpis* is the principal hematophagous vector. High infection rates occur in areas of environmental degradation and human migration associated with urbanization. Dogs are the principal reservoir for the parasite, playing a central role in transmission to humans, and present a serious public health concern [1]. The main histological alterations observed in dogs with visceral leishmaniasis are hypertrophy and hyperplasia of cells of the mononuclear phagocyte system in spleen, lymph nodes, liver, and bone marrow; a chronic inflammation of the skin; granulomatous inflammatory reactions in liver and spleen; interstitial pneumonitis; and glomerulonephritis with or without nephrotic syndrome. Gastrointestinal tract (GIT) disorders occur in human visceral leishmaniasis (HVL) and in canine natural and experimentally induced infections [2].

GIT disorders occur in response to CVL as a result of natural infection [2-8] and experimental infection [9,10]. Researchers have described a severe chronic inflammatory process in the mucosa and submucosa, predominantly localized in the lamina propria (LP) of the intestinal mucosa (cecum and colon), which is associated with higher levels of parasitism when compared with other GIT segments. Mononuclear exudates are composed of plasma cells, macrophages, and lymphocytes, with few neutrophils and eosinophils. Adamama-Moraitou et al. [8] described pyogranuloma formation. In a recent study (unpublished results), we investigated 20 dogs naturally infected with *L. infantum* from an endemic metropolitan area of Belo Horizonte, Minas Gerais, Brazil, and observed high parasite burden throughout the GIT mucosa (predominantly in the colon) of symptomatic and asymptomatic dogs without marked pathological alterations (Unpublished information).

The detection of specific microorganisms by innate immune cells is mediated by pattern recognition receptors (PRRs), germ line-encoded receptors that recognize microbial structures referred to as pathogen-associated molecular patterns. The potential contribution of Toll-like receptors (TLR) in fighting parasitic infections has gained attention in the past decade. TLRs are essential PRRs that mediate the recognition of microbial structures and induce inflammatory and adaptive responses. Several studies have shown the recognition of *Leishmania*-derived molecules by different TLRs [11,12]. During the past decade, it has become clear that a family of non-clonal, germline-encoded PRRs, the mammalian TLRs, provides the innate immune system with considerable specificity for a spectrum of microbial pathogens [13,14]. Recently, TLRs have been demonstrated to be essential for activation of immune cells, including macrophages and dendritic cells, through the recognition of microbial components including lipopolysaccharide (LPS) from gram-negative bacteria, lipoprotheic acid, lipoproteins produced by all bacterial pathogens, and peptidoglycan, the main stimulatory component of gram-positive bacteria [15-18]. Thirteen mammalian TLRs have been described, with ten expressed in humans, each responsible for the recognition of distinct, invariant microbial structures, not expressed by the host, and known as pathogen associated molecular patterns (PAMPs) [12].

The effector site in the intestine is the mucosal epithelium that underlies the LP in the small and large intestines. Homeostasis in the gut seems to be a result of precise interaction among activated T cells, plasma cells, mast cells, dendritic cells, and macrophages, characterizing a state of physiological inflammation. However, microbiota and dietary antigens are continuously absorbed, reaching the LP, Peyer´s patches, and isolated lymphoid follicles in the mucosal layer of the intestine as well as the mesenteric lymph nodes. This gastrointestinal-associated lymphoid tissue (GALT) constitutes the largest and most complex immunologic organ of the body and must be capable of mounting protective immune responses to pathogens while maintaining tolerance to harmless environmental antigens such as food and commensal microbes [19,20].

The GIT consists of the small intestine (duodenum, jejunum, and ileum), and the large intestine (cecum and colon). As with other cells of the vertebrate body, intestinal cells can express all TLR types. Normally, intestinal epithelium is not inflamed despite close contact with a high density of commensal organisms that could elicit inflammation [19,21,22]. The differential expression of TLRs in distinct anatomical compartments of the gut epithelium of the GIT is likely to contribute to avoiding over-stimulation [14,20,23].

Several non-TLR chains cooperate with TLRs to recognize PAMPs. CD14 and the integrin-like CD11b/CD18 receptors (CR3 - complement receptor type 3 or Mac-1) are examples of such receptors [24]. In a previous study conducted by our group, peripheral blood mononuclear cells of infected dogs were evaluated using surface receptors including CD14, CD11b, TLR2, and MHCII. Dogs bearing CD14 monocytes with higher expression of CD11b had a lower parasite load in ear tissue. These parasites were unable to infect phlebotomines (IHQ-/XENO-). Conversely, dogs with lower expression of CD11b in CD14 monocytes had higher parasite load in the ear tissue, and these parasites were able to infect phlebotomines (IHQ+/XENO+). Higher values of CD11b^+^/TLR2^+^ and CD11b^+^/MHCII^+^ were obtained from dogs with IHQ-/XENO- than dogs with IHQ+/XENO+. These data lead to the conclusion that IHQ-/XENO- dogs are either more resistant to infection or better able to mount a cellular immune response essential for *Leishmania* tissue clearance [25]. Recent data also demonstrated that TLR9 plays a role in the recognition of *Leishmania*[12], although several questions concerning the role of TLR2 and TLR9 in *L. infantum* infection of the canine GIT remain to be answered, considering that the GIT microflora has a marked gradient and plays an important role in maintenance and regulation of gut immunity. Since TLR recognition is often associated with the production of pro-inflammatory cytokines and with the generation of additional effector molecules, it is important to determine the implications of TLR activation during *Leishmania* infection. A few *Leishmania*-derived molecules have been reported to activate TLRs, and the majority of the studies to date have focused on the activation of TLR2 and TLR9. Thus the aim of this study was to investigate the correlation between parasite load and expression of TLR2 and TLR9 by LP myeloid cells of the jejunum and colon co-expressing CD11b, CD11c, and CD14 receptors.

## Results

### Clinical examinations

**Figure 1:**
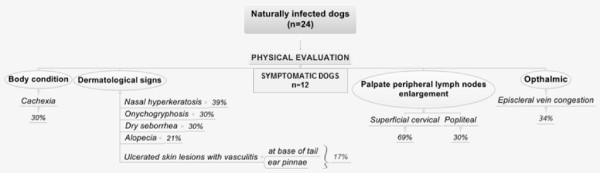
**Clinical findings of twenty-four dogs naturally infected with *Leishmania (L.) infantum*, Belo Horizonte, Minas Gerais, Brazil.**

Physical evaluation of all animals (n = 24) was carried out before necropsy. All dogs exhibiting external clinical signs presented with dermatological symptoms. A single symptomatic animal was observed to experience pain on abdomen palpation. The clinical lesions observed in symptomatic dogs were: superficial cervical (69%) and popliteal (30%) lymph node enlargement; nasal hyperceratosis (39%); ocular congestion (34%); onychogryphosis (30%); cachexia (30%); dry seborrhea (30%); alopecia (21%); vasculitis of the tail (17%); and ulcerated lesions, mainly in the extremity of the ear (17%) (Figure 1). This study considered dogs that presented at least one classical clinical sign of disease to be symptomatic. All symptomatic dogs exhibited more than one clinical sign of visceral disease. Animals were considered asymptomatic if they showed no clinical sign of leishmaniasis. During necropsy, no severe macroscopic or microscopic lesion in the mucosa of the GIT was evident. In approximately 25% of the dogs, the mucosa was hyperemic; although no other severe macroscopic or microscopic alteration was found (focal hyperemia was observed but no hemorrhages, ulcers, or pyogenic granulomas).

### Histological analysis

An increase of cellularity of LP was evident in all samples of infected dogs than controls. The presence of mononuclear cells was more frequent in the mucosal and submucosal layers of jejunum and colon tissue fragments of infected dogs than controls, regardless of their clinical status. Histological analysis revealed higher levels of cellularity (plasma cells, macrophages, and lymphocytes) in the LP of jejunum and colon in infected dogs than in control dogs. Macrophages frequently presented with atypical morphology characterized by pale and abundant cytoplasm and a less dense nucleus than is typically seen. This was associated with areas of concentrations of mononuclear cells, but no typical granulomatous reaction was evident in the LP. A small number of neutrophils and eosinophils were observed associated with a diffuse mononuclear LP cellularity.

Morphometric analysis of LP cells in jejunum and colon tissue of infected dogs demonstrated no significant difference between jejunum and colon. There were no significant differences in LP cellularity of infected asymptomatic versus symptomatic dogs. In liver, spleen, lymph nodes, and bone marrow of infected dogs, a pattern of injury was found similar to that described by Tafuri et al. [26]. In liver, the most frequent alterations were granulomatous inflammation, hydropic and adipose degeneration, and hemosiderosis. Hemorrhage, plasmacytosis, congestion, and capsule inflammation were observed in spleen. Lymph nodes showed plasmacytosis and subcapsular and cortical follicle hyperplasia. *Leishmania* amastigotes were seen in these organs.

**Figure 2:**
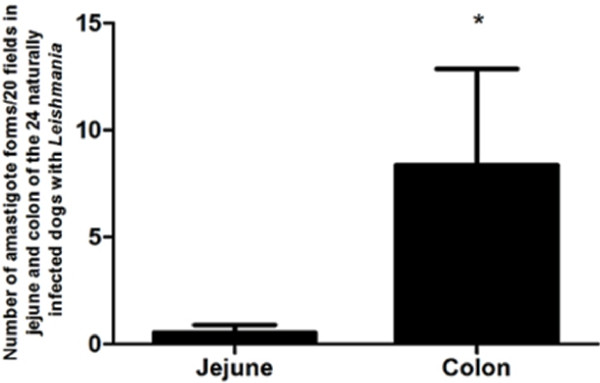
**Morphometric analysis of the parasite tissue burden in jejunum and colon of twenty four naturally infected dogs.** Number of amastigotes/20 fields *colon harbors more parasites than jejunum p = 0.0231.

Amastigotes of *Leishmania* were present in all GIT layers, particularly inside macrophages of the LP. Despite the parasite load, no erosion or ulcers were evident in the epithelial mucosa layers or the glands, and morphometric analysis of the parasite burden of canine jejunum demonstrate a significant difference from that of the colon, with the colon harboring more parasites than the jejunum (p = 0.0231; Figure 2). No difference was observed between symptomatic and asymptomatic dogs. A positive correlation of inflammatory cells with parasite load was observed in jejunum (r^2^ = 0.3852) and colon (r^2^ = 0.2544).

**Figure 3:**
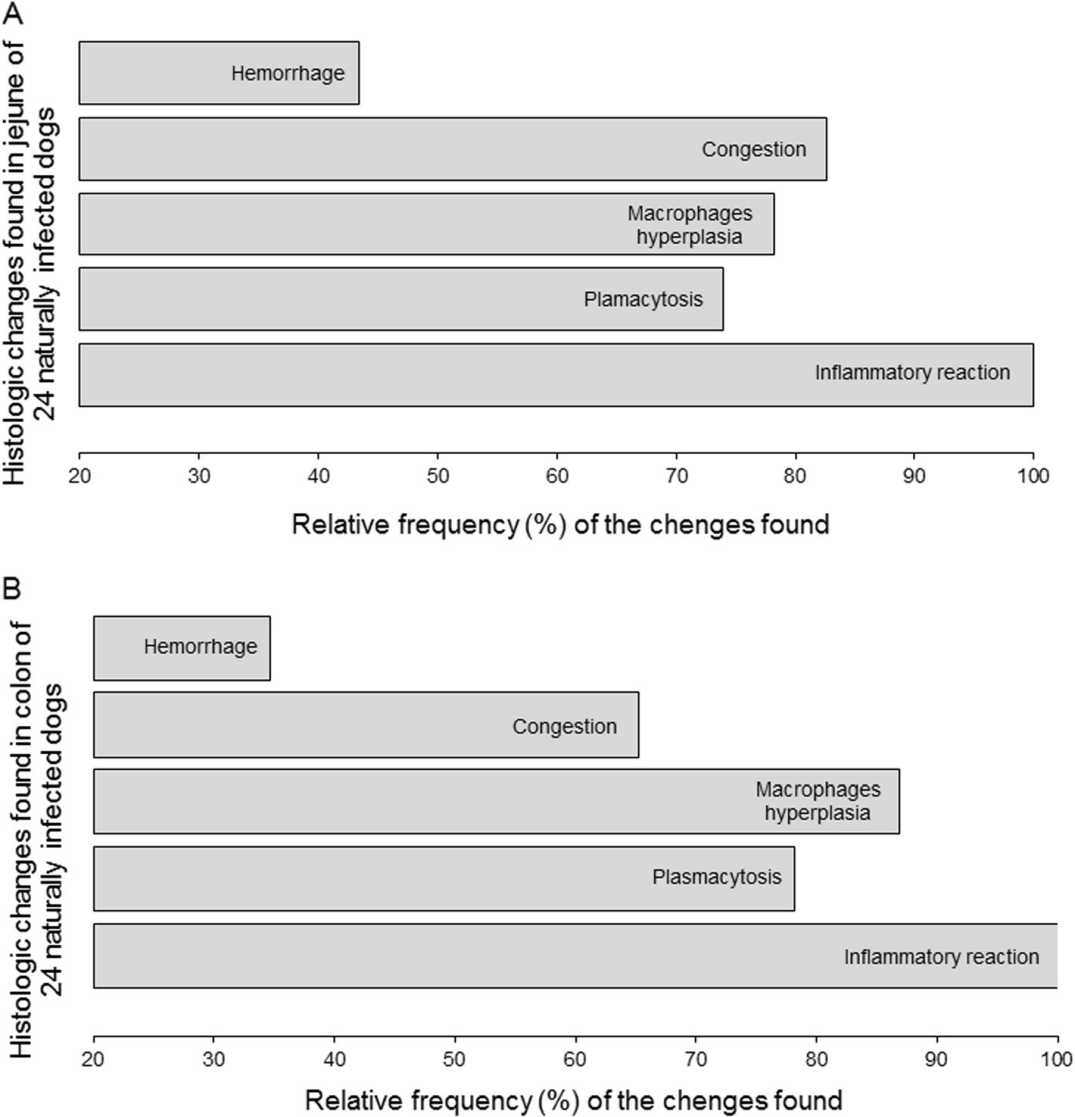
**Semi quantitative analysis.** Histopathological analysis of the images analyzed using software, viewed on video screen and sent to a computer-assisted image analysis system (Kontron Elektronic/Carl Zeiss, Germany): **(A)** Intensity of jejunum alterations; **(B)** intensity of colon alterations.

Semi-quantitative histological analysis of all samples of jejunum and colon was conducted by optical microscopy. Frequency of histological changes in jejunum and colon were hemorrhage (43%, 34%, respectively), congestion (82% and 65%), macrophage hyperplasia (78%, 86%), plasmocytosis (73%, 78%) and high levels of mononuclear cellularity (100%,100%) (Figures 3A and B).

### Myeloid cell expression of CD11b, CD14, CD11c, TLR2, and TLR9 in the mucosa of the colon and jejunum

**Figure 4:**
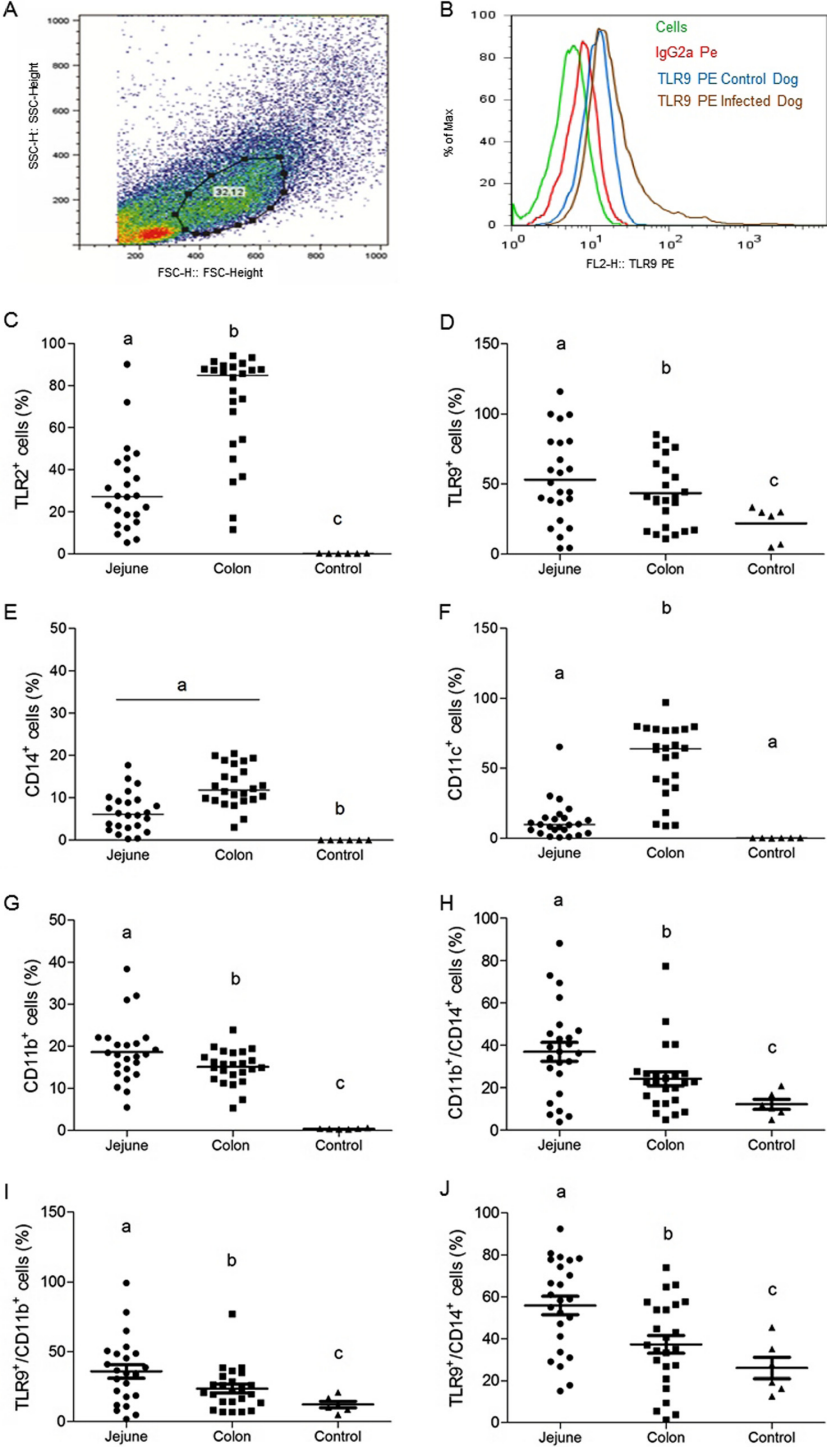
**Representative gating strategies demonstrating fluorescence profiles of cells in jejunum and colon lamina propria cells in dogs naturally infected with *Leishmania*; (A) macrophages were identified on the basis of their specific forward (FSC) and side (SSC) light-scattering properties (B) isotype control cutoff; (C) frequency of TLR2**^**+**^**; (D) frequency of TLR9**^**+**^**; (E) frequency of CD14**^**+**^**; (F) frequency of CD11c**^**+**^**; (G) frequency of CD11b**^**+**^**; (H) frequency of CD11b**^**+**^**/CD14**^**+**^**; (I) frequency of TLR9**^**+**^**/CD11b**^**+**^**; (J) frequency of TLR9**^**+**^**/CD14**^**+**^**.**

Lamina propria cells from colon and jejunum were analyzed by flow cytometry. Myeloid cells were identified based on specific forward (FSC) and side (SSC) light-scattering properties (Figure 4A), and expression of receptors was assessed using monoclonal antibodies and isotype controls (Figure 4B). A higher frequency of TLR2^+^ cells was demonstrated in colon compared to jejunum (p = 0.0001) (Figure 4C). In contrast, a numbers of TLR9^+^ cells were evident in jejunum (p = 0.0364) (Figure 4D). There was no difference in frequency of colon and jejunum cells expressing CD14 (Figure 4E). CD11c^+^ cells were elevated in colon (p = 0.001) (Figure 4F), whereas CD11b^+^ cells and CD11b^+^/CD14^+^ cells predominated in jejunum (p = 0.005) (Figure 4G and H). Frequency of CD11b^+^ cells and CD11b^+^/CD14^+^ cells expressing TLR9 was elevated in jejunum than colon (Figure 4I and J). Jejunum and colon segments of symptomatic and asymptomatic dogs showed no significant differences in the frequency of studied receptors, with the exception of a CD14^+^ subset which was present at higher levels in symptomatic dogs (p = 0.0247 in jejunum and p = 0.0245 in colon). A positive correlation between inflammatory cells and parasite load was observed in jejunum (r^2^ = 0.3852) and colon (r^2^ = 0.2544). There was a low positive correlation between parasitism and receptors studied, with an average r^2^ = 0.1412 in jejunum and r^2^ = 0.0497 in colon. A higher frequency these receptors were demonstrated in infected dogs compared to control dogs.

### Cytokines

**Figure 5:**
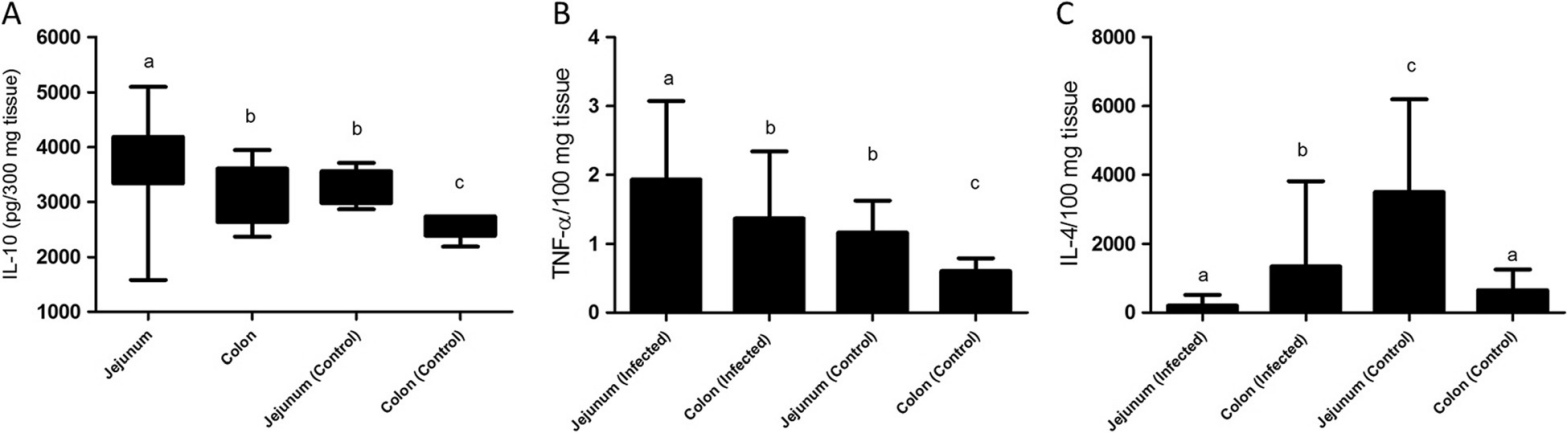
**Cytokine expression in jejunum and colon of dogs infected with *Leishmania infantum chagasi*: (A) IL-10 differs in colon and jejunum, p = 0.0032, (B) IL-4 differs in colon and jejunum, p = 0.0002; (C) TNF-α differs in jejunum and colon, p = 0.0043.** Different letters show significant differences between the groups.

Despite the negative ELISA serum results for interleukin 4 (IL-4), interleukin 10 (IL-10) and tumoral necrotic factor-alpha (TNF-α) cytokines were detected in jejunum and colon tissue samples from all infected animals. IL-10 and TNF-α were higher in jejunum than in colon of infected animals (p = 0.0032 and p = 0.0002, respectively). IL-4 was higher in colon than jejunum of infected animals (p = 0.0043) (Figure 5A-C). A higher expression these cytokines were demonstrated in infected dogs compared to uninfected dogs.

## Discussion

Histological analysis of all segments of GIT of infected dogs revealed intense cellularity in the LP of the mucosal layer. These cells were represented by plasma cells, macrophages, and lymphocytes. Macrophages were hypertrophic and frequently infected by amastigotes of *Leishmania*. Morphometric analysis of the LP cellularity demonstrated comparable numbers of cells in the jejunum and colon, but the parasite load was higher in colon. Despite the parasite burden throughout the GIT mucosa, no ulcer, focus of hemorrhage, or marked pathological alterations were observed. These results are in accordance with Silva et al. [7] and Pinto et al. [1]. However, Adamama-Moraitou et al. [8], using colonoscopy, and immunohistochemistry observed mild erosive colonic mucosa in eight of 31(25.8%) symptomatic dogs naturally infected with *L. infantum*. The dogs included in their study did not present a history or clinical evidence of overt colitis. These authors found pyogranulomatous formation to be the most common histological feature in the colonic mucosa. It has been suggested that the granulomatous inflammatory reaction is directly related to a low parasite tissue burden [27]. In the current study, immunohistochemical morphometric analysis showed a higher parasite load in colon than in jejunum, which is in accordance with the literature [1,2,6,7,9,15,28]. A positive correlation between parasite load and cellularity in the LP was evident (r^2^ = 0.3852).

The presence of excessive cellular infiltrate in the LP has been associated with microflora; parasite load of biological agents including viruses, fungi and protozoa; and the immunologic status of the host. Gastrointestinal mucosa is home to the largest number of leukocytes in the body, and is the site where these cells encounter abundant exogenous stimuli. Despite this constant immunological stimulation, immune responses in the intestine remain in a state of controlled inflammation: the physiological inflammatory status [19,22,29]. Emerging evidence indicates that TLRs are involved in the immunological homeostasis of GIT. Crypt epithelial cells express TLR2 and TLR4, whereas a mature intestinal epithelial cell (IEC) expresses only TLR3 [30]. Since crypt epithelial cells do not come into direct contact with commensal bacteria, expression of TLR2 and TLR4 should not be detrimental to the host. TLR3 expression in the intestinal lumen is also not damaging, since the TLR3 ligand, viral double-stranded RNA, is not naturally present in the gut microbiota. Inflammation associated with the expression of TLR9 by IEC is minimized, since this receptor is unresponsive to CpG due to the presence in the gut mucosa of TLR-antagonists that can suppress the activation of TLR9 [31-33]. In addition, resident macrophages in intestinal LP seem to play an important role in GIT mucosa homeostasis. Resident macrophages of the jejunum and colon are located in the LP just below the mucosal layer [34] and are present in the sub-epithelial dome region of Peyer´s patches. Recent studies have demonstrated that human colonic macrophages express the human macrophage marker, macrosialin (CD68), low levels of the integrins CD11b (CR3) and CD11c (CR4), and MHC class II antigens [35]. Intestinal macrophages lack CD14 receptors, a glycosilphosphatidyl inositol (GPI)-linked glycoprotein that forms part of the high affinity complex essential for LPS recognition expression. Intestinal macrophages differ from their peripheral counterparts, as they originate from circulating monocytes that express a range of functional TLRs, integrins CD11a, b, c/CD18, and CD14 positive receptors [16,35]. Therefore, it appears that intestinal macrophages down-regulate TLRs upon arrival in the mucosa, although the underlying mechanism is unknown [22].

Canine TLR2 has been sequenced, with moderate expression of TLR2 mRNA detected in the small and large intestine [36], but no TLR9 mRNA was detected using conventional PCR [37]. However, Burgener et al. [38], using RT-PCR, detected mRNA coding for TLR2 and TLR9 in the duodenum and colon of dogs with GIT chronic disorders. These authors found higher TLR2 expression in the duodenum than the in the colon. They also described TLR9 expression in all segments of GIT, with a significantly higher expression in ileum than in stomach, jejunum, or colon. In our study of dogs naturally infected with *Leishmania*, we found lower frequencies of TLR2^+^ cells and higher frequencies of TLR9^+^ cells in jejunum than in colon.

Little information is available on TLRs in the canine intestine, although it is known that TLR2 and TLR4 are expressed at low levels in non-stimulated canine primary colon epithelial cells and can be upregulated in response to challenge by their respective agonists, peptidoglycan and lipopolysaccharide [39]. Upregulation of TLRs in canine macrophages obtained from the LP of jejunum and colon could be explained either by recruitment of inflammatory cells expressing TLRs or by proliferation of resident macrophages induced by *Leishmania* antigens. *Leishmania* reaches intestinal LP via blood or lymphatic vessels, and TLR2 and TLR9 on the macrophage surface can interact with *Leishmania*[40,41]. de Veer et al. [11] showed that TLR2 activates NF-κB mediated by LPG. Unlike TLR2, TLR9 appears to be intracellular, but it can be upregulated and expressed on the cell surface, where it can participate in cellular activation and lead to inflammation or tolerance in mice. In humans, TLR9 has been implicated in NK cell activation being essential for IL-12 production by dendritic cells [41]. In equines, an enhancing effect of TLR9 ligation to antigen-specific Th1 immune responses to intracellular pathogens has been described by Zhang et al. [42]. Similar results were observed in dogs with GIT disorders [38]. We found an association between a lower parasite burden in the jejunum and a higher frequency of TLR9^+^ cells, suggesting TLR9 correlation with parasite clearance. On the other hand, TLR2 has been described as associated with TGF-beta production and regulatory function, rather than inflammatory activity of macrophages [43]. TLR2^+^ cells were more abundant in colonic tissue of infected dogs. Vivarini et al. (2011) [44] suggest that TLR2 plays a role in facilitating the establishment of the disease, depending on the *Leishmania* species involved.

We found a correlation between higher frequencies of CD11b^+^ and CD14^+^ macrophages expressing TLR9 in the jejunum as compared to colon in infected dogs. In canine visceral leishmaniasis, peripheral monocytes TLR2^+^/CD11b^+^/CD14^+^[25] and hepatic macrophages CD11b^+^/CD18^+^[45] seem to be associated with lower tissue parasite load. In contrast, splenic macrophages splenic cells (monocyte-macrophages) might serve to perpetuate the intracellular infection [45,46]. Thus, as reported in the literature, there is a compartmentalization of the immune response against *Leishmania* in different organs [46]. According to Ettinger and Wilson [47], the initial encounter between *L. infantum* and cells of the innate and adaptive immune systems stimulates primarily type 1 immune cytokine responses (IFN-γ, IL-6, IL-1α, IL-1β). CD11b and CD14 are important macrophage receptors involved in this initial interaction and the production of pro-inflammatory cytokines [11]. However, resident intestine macrophages do not express CD11b and CD14 antigens in healthy GIT. Therefore, the presence of *Leishmania* in LP could modify the LP environmental homeostasis, generating mediators responsible for chemotaxis of circulating monocytes to the mucosa. These recently recruited monocytes may not remain in the location for a period of time sufficient for adaptive differentiation to occur, or it may be that *Leishmania* presence heightens the level of immune-stimulatory signals, overcoming the usual regulatory processes [22,23]. Parasitic infections can also provide stimulation for the expression of TLR9 by the macrophages, as reported in dogs with chronic inflammatory disorders [38]. Therefore, the correlation between the frequencies of CD11b^+^/CD14^+^ cell expressing TLR9 in the jejunum with the lower parasite load at this site suggests that TLR9 may play an effective role in inducing a cellular immune response against *Leishmania* in macrophages. TLR9 stimulation could lead to protective immunity against the parasite. However, results should be interpreted with caution; more research needs to be done to correlate TLR expression with a potential role for those receptors in generating responses in leishmaniasis. Increased expression of TLR2 in colon and TLR9 in jejunum may occur as a consequence of the inflammatory environment of the GIT, making it difficult to evaluate the extent to which *Leishmania* is implicated in the activation of TLRs.

Infected dogs showed higher IL-10, IL-4 e TNF-α levels than controls, regardless of clinical status. However, TNF- α and IL-10 was higher in jejunum, with a lower parasite load, than in colon. In contrast, IL-4 was higher in colon than jejunum. TNF- α has been implicated as an anti-*Leishmania* proliferation cytokine, with IL-4 and IL-10 being related to the susceptibility to visceral disease. Thus, our results for TNF- α and IL-4 are in accordance with the literature regarding their role in canine visceral leishmaniasis. However, the higher level of IL-10 found in jejunum could be a conflicting result, if we consider IL-10 a cytokine related to the progression of disease. Higher levels of IL-10 in jejunum than colon associated with a lower parasite load may be explained by TNF- α ability to induce T cells and regulatory T cells responsible for IL-10 production (26).

## Conclusion

There was no correlation between clinical signs and pathological changes and immunological and parasitological findings in the gastrointestinal tract in canine visceral leishmaniasis. However, distinct segments of GIT presented different immunological and parasitological responses. Jejunum showed a lower parasite load with increased frequency and expression of CD11b, TLR9, CD14/CD11b/TLR9 receptors and IL-10 and TNF-α cytokines. Conversely, the colon showed a higher parasite load along with increased frequency and expression of TLR2, CD11c receptors, and IL-4 cytokine. Thus, *Leishmania infantum* is able to interfere in jejunum increased expression of TLR2, TLR9, CD11b, CD14, CD14/CD11b/TLR9 receptors, IL-10, and TNF-α; and in colon increased expression of CD11c, TLR2, TLR9, CD11b, CD14 e, CD14/CD11b/TLR9 receptors, IL-10, and TNF-α.

## Methods

### Animals

This study received the approval of the CETEA/UFMG (Comitê de Ética em Experimentação Animal/Universidade Federal de Minas Gerais), protocol 257/2008. All procedures involving animals were conducted according to guidelines of the Colégio Brasileiro de Experimentação Animal (COBEA).

### Uninfected dogs

Six dogs (control group) of unknown age, negative for *Leishmania* infection were obtained from the Control Zoonosis Center of the Municipality of Carandaí, Belo Horizonte Metropolitan area, Minas Gerais (MG) State, Brazil. In all tests the dogs (n = 6) had negative results. This protocol received the approval of the CETEA/UFMG (protocol 139/2012).

### Naturally infected dogs

Twenty-four adult mongrels dogs, naturally infected with *L. infantum,* were obtained from the Zoonosis Center of metropolitan area of Belo Horizonte (Municipality of Ribeirão das Neves, state of Minas Gerais, Brazil). Prior to inclusion in the study, dogs received anti-helmintic and anti-ectoparasitic treatment and were immunized against parvovirus, distemper, leptospirosis, para-influenza, and hepatitis (HTLP5CV-L vaccine PfizerH). They were maintained for 30 days in kennels at the Department of Parasitology of Instituto de Ciências Biológicas (ICB), Universidade Federal de Minas Gerais (UFMG), Belo Horizonte, MG, Brasil. Commercial dog food and water were provided *ad libitum*. Infection was confirmed by serological tests, polymerase chain reaction, and immunohistochemistry. Dogs (n = 24) showed positive results in all tests.

### Clinical examination

Clinical examinations were carried out on 24 infected dogs to evaluate body condition, vital signs, palpable peripheral lymph nodes (cervical and popliteal), and dermatological signs. Animals were categorized according to the presence of clinical signs of visceral leishmaniasis, and infected dogs were divided into two groups: Group I, asymptomatic, consisted of 12 dogs showing no classic symptoms of leishmaniasis; Group II, symptomatic was 12 dogs that exhibited classic signs of CVL including lymphadenopathy, cutaneous alterations (alopecia, dry exfoliative dermatitis or ulcers), onychogryphosis, keratoconjunctivitis, and cachexia.

### Indirect immunofluorescence antibody test (IFAT)

IFAT was used to detect *Leishmania* antibodies. Antigen was prepared from *L. infantum* MHOM/BR/1967/BH46 promastigotes and fixed on slides. Serum samples were diluted at 1:40 in phosphate buffered saline (PBS), and 25 mL was placed on demarcated regions of the slide. The slide was incubated in a humid chamber at 37°C for 30 min, washed with PBS, and dried at room temperature. Then 25 mL of a commercially available fluorescein-conjugated anti-dog IgG (Bethyl® Laboratories, Montgomery, TX, USA) diluted at 1:1500 in PBS containing 2% Tween (Tween 80, Merck®, Germany) was added to each demarcated region of the slide, followed by incubation, washing, and drying. Samples presenting fluorescence at 1:40 dilution were considered positive [25].

### Enzyme linked immunosorbent assay (ELISA)

Determination of anti-*Leishmania* IgG was carried out using standard ELISA. *Leishmania* soluble antigen was derived from *L. chagasi* strain MHOM/BR/1967/BH46 promastigote forms ruptured ultrasonically. Aliquots (100 μL) of soluble antigen dissolved in 0.05 M carbonate buffer (pH 9.6) to a final concentration of 2 mg/mL were transferred to individual wells of a 96-well microplate and incubated overnight at 4°C. Coated wells were washed five times with PBS containing 0.2% Tween-20, and antigenic sites were saturated with 150 μL PBS containing 0.2% Tween-20 and 2% casein (Sigma®, St Louis, MO, USA; product # C0376) for 30 min at 37°C, washed three times with PBS, and a 100 μL-aliquot of serum sample (diluted 1:400) was placed into each well. Plates were incubated for 45 min at 37°C, washed five times with PBS, and a 100 μL aliquot of diluted enzyme-labelled immunoglobulin was added to each well. The titer of rabbit-anti-canine-IgG conjugates (Sigma®; product # A6792) was 1/10 000. Following incubation for 45 min at 37°C, plates were washed five times with PBS and a 100 μL aliquot of 4% (w/v) ortho-phenylenediamine in phosphate/citrate buffer (pH 5) containing 4 μL of 30 (v/v) hydrogen peroxide was added to each well. Reaction mixture was incubated at 37°C for 10 min. Reaction was stopped by addition of 25 μL 2M sulphuric acid to the well, and absorbance was measured at 492 nm using a BioRad (São Paulo, SP, Brazil) model 550 ELISA reader. The cut-off point was the mean absorbance reading of the VL-negative controls plus two standard deviations [25].

### Polymerase chain reaction (PCR)

Specific PCR carried out on ear skin samples was used to confirm that animals were positive to *L. infantum*. Twenty-five mg ear skin samples were extracted using the DNeasyH Blood & Tissue Kit (Qiagen Inc., Valencia, CA, USA) according to the manufacturer’s instructions. Oligonucleotide primers LV1 (59 ACGAGGTCAGCTCCACTCC 39) and LV2 (59 CTGCAACGCCTGTGTCTACG 39) used for DNA amplification were specific for a repetitive DNA sequence in *L. infantum*. Amplified PCR products were analyzed on 5% silver-stained polyacrylamide gels [39]. All dogs were positive. Six dogs negative for *Leishmania* infection were used as controls.

### Immunohistochemical (IHC)

As well as for diagnosis, the immunohistochemistry reaction was used to quantify the parasite load in the studied tissue. Deparaffinized slides were hydrated and incubated with 4% hydrogen peroxide (30 v/v) in PBS 10%, pH 7.2, followed by incubation with normal goat serum (diluted 1:50). Heterologous immune sera from dogs naturally infected with *L. infantum* (diluted 1:100 in 0.01 M PBS) were used as a primary cross-reactive antibody as described by Tafuri et al. [48]. Slides were incubated for 18–22 h at 4°C in a humid chamber, washed with PBS, incubated with biotinylated goat anti-mouse and anti-rabbit (Link-DAKO, LSAB2 kit, California, USA), washed once with PBS, and incubated with streptoavidin-peroxidase complex (Link-DAKO, LSAB2 kit, California, USA) for 20 min at room temperature. Reaction was developed with 0.024% diaminobenzidine (DAB: Sigma, Saint Louis, USA) and 0.16% hydrogen peroxide (40 v/v). Slides were counterstained with Harris's hematoxylin, dehydrated, cleared, and mounted with coverslips. A quantitative study was carried out for optical microscopy and morphometrical analysis. Twenty images were randomly chosen and used to count immunolabeled amastigotes. Images were analyzed as described in the previous section. They were obtained using software, viewed on a computer video screen, and transferred to a computer-assisted image analysis system (Kontron Elektronic/Carl Zeiss, Germany), as previously presented [45].

In Brazil, animals with canine visceral leishmaniasis are usually not treated, as animal treatment is prohibited by Brazilian Health Ministry (Portaria Interministerial 1.426 de 11 July 2008) to avoid creating reservoirs of the parasite. After infection confirmation, animals were euthanized using 2.5% (1.0 ml/kg) thiopental (intravenous) and T61™ (0.3 ml/kg) [1].

### Histopathology

During necropsy, tissue impression smears of skin, lymph node, liver, and spleen were obtained to evaluate *Leishmania* infection. In addition, samples of jejunum and colon segments were collected, fixed in 10% buffered formalin, dehydrated, cleared, embedded in paraffin, sectioned (3 μm thick), and stained with hematoxylin and eosin for histopathological studies. Each GIT segment was macroscopically sliced into three fragments. Therefore, each histological slide contained three longitudinal sections. Blind histological analysis of slides was carried out by a minimum of two pathologists.

For the histological evaluation of a chronic inflammatory reaction in jejunum and colon, we carried out semi-quantitative and quantitative analysis. For semi-quantitative analysis, inflammatory mononuclear cells were counted using ocular analysis and rated: (1) Slight: 1–9 cells per field/20 fields; (2) moderate: 10–30 cells per field/20 fields; (3) intense: > 30 cells per field/20 fields. For quantitative analysis, images were analyzed using software, viewed on a computer video screen, and transferred to a computer-assisted image analysis system (Kontron Elektronic/Carl Zeiss, Germany) [49]. Under microscopic analysis, a minimum of 20 fields for each slide mounted with paraffinized GIT segment fragments was counted. Horizontal and vertical movements (with the microscope stage – XY translation) were carried out to avoid overlapping fields (repetitions). Therefore, we used the word “random” to indicate that we took care not to analyze fields in duplicate. This histomorphometric study used 20 randomly chosen images from histological slides of organ tissue fragments to assess the number of immunolabeled amastigotes using a Kontron Elektronick/Carl Zeiss image analyzer (KS300 software) and an Axiolab light microscope (Zeiss) with a ×440 resolution [50].

### Flow cytometry

To isolate normal lamina propria cells, the entire length of the jejunum and the colon was opened longitudinally, washed with PBS, and cut into small pieces. The dissected mucosa was incubated with Ca^2+^, Mg^2+^-free Hank’s Buffered Salt Solution (HBSS) for 60 min under slow agitation with HBSS changes every 10 min. Fragments were incubated in Ca^2+^, Mg^2+^-free HBSS containing 1 mM DL-Dithiothreitol (DTT® - Sigma-Aldrich) twice for 30 min under slow agitation at 37°C to remove mucus, and treated with 0.11 mg/mL collagenase II for 4 h on a slow-moving shaker at 37°C with the supernatant collected every 45 min. Supernatants were centrifuged at 1300 rpm for 10 min and subjected to erythrocyte lysis with 9 mL distilled water for 30 sec. 1 mL of concentrated PBS was added to stop lysis, and the solution was centrifuged for 10 min at 1300 rpm at 4°C. Pellets were resuspended in 950 μl Roswell Park Memorial Institute (RPMI) medium. Final volumes were adjusted to 1 mL, and concentrations adjusted to 1 × 10^7^ cells/mL. To isolate cells from cervical and mesenteric lymph nodes, tissues were macerated in 2 mL of RPMI and centrifuged for 10 min at 1300 rpm at 4°C. Macerated tissue was resuspended in 9 mL distilled water and 1mL PBS concentrate and centrifuged for 10 min at 1300 rpm at 4°C. This procedure was performed twice to lyse erythrocytes. Pellets were resuspended in 950 μL complete RPMI medium and 50 μL (5%) containing serum from non-infected dogs. The final volume was adjusted to 1 mL, and the concentration was adjusted to 1 × 10^7^ cells/mL as described by Figueiredo et al. [51].

A 20 μL cell suspension was incubated in 96-well U-bottom plates (Limbro Biomedicals®175, Aurora, OH, USA) at 4°C for 30 min in darkness in a 20 μL solution containing monoclonal antibodies (mAbs) to cell surface and intracellular markers diluted to 10% in PBS. Non-conjugated, purified antibodies were conjugated using a Zenon tricolor kit (Molecular Probes – Z-25080) as instructed by the manufacturer. After staining, samples were washed with 0.1% Na_3_N in PBS, fixed with 200 μL 2% formaldehyde in PBS, and stored at 4°C until analysis by a flow cytometer (FACSVantage, Becton & Dickinson, San Jose, CA, USA). For intracytoplasmic marking, the protocol described by Figueiredo et al. [51] was followed.

Cells were analyzed by an analytical flow cytometer equipped with a laser emitting at 488 nm (FACSVantage, Becton-DickinsonH, San Diego, CA, USA). Whole cells were distinguished from fragments by gating based on the forward and side scatter signals, and a minimal of 40000 events were acquired for each sample [25,51]. Data were analyzed using the program FlowJo® (Tree Star. Inc., Ashland, OR, USA). The phenotypic aspects of lamina propria cells were expressed as percent cells expressing a given phenotypic marker, using cell and isotype control cut-off, with a bimodal distribution. Fluorochrome-labeled antibodies used for staining were: anti-humanTLR-9 1:30 (mouse, clone IMG-305A, PE, Imgenex®, USA); anti human-TLR-2 1:20 (mouse, clone 2B4A, PE, SourthernBiotech®, USA); anti-canine CD11b 1:20 (mouse, clone MCA 1777S, FITC, Serotec® USA); anti-human CD14 1:20 (mouse, clone MCA1568C, CY, Serotec® USA) [25] and anti-CD11c 1:20 (FITC, Serotec® USA).

### ELISA of Serum and Jejunum and Colon Extracts

Serum and extracts of tissue samples from jejunum and colon of all animals were collected for quantitative analysis of TNF-α, IL-4, and IL-10 cytokines by capture-ELISA. 100 mg of tissue, ground in a tissue homogenizer (T10 Basic Ultra Turrax Disperser IKA®) for approximately 5 min in 2 ml of RPMI-1640 media (Sigma, St. Louis, MO, USA), pH 7.2, was used. The resulting homogenate was centrifuged at 10 000 rpm for 15 min at 4 C. Eppendorf 5810 R, Hamburg, Germany). TNF-α, IL-4, and IL-10 quantitative analysis were carried out using anti-canine IL-4 monoclonal antibody (Canine IL-4, Mouse IgG1 cod#MAB 754, R&D systems), IL-10 (Canine IL-10, Mouse IgG1 cod#MAB 7352, R&D systems), and TNF-α (Canine TNF alfa, Mouse IgG1, cod#MAB 1507, R&D systems).

### Statistical analysis

Parametric and non-parametric nature of the data was evaluated. Friedman and Mann Whitney tests were used for statistical analysis. Friedman test was used to compare each segment of the GIT within each group of dogs, and the Mann Whitney test was used to compare GIT segments between asymptomatic and symptomatic dogs using Graph Pad InStat and Prism5. Pearson and Spearman tests were used for correlation analysis. In all cases, statistical differences were considered significant when the probabilities were p < 0.05.

## Abbreviations

TLR: Toll-like receptor; CVL: Canine visceral leishmaniasis; GIT: Gastrointestinal tract; HVL: Human visceral leishmaniasis; PRRs: Pattern recognition receptors; LPS: Lipopolysaccharide; PAMPs: Pathogen associated molecular patterns; GALT: Gastrointestinal-associated lymphoid tissue; CETEA: Comitê de Ética em Experimentação Animal; UFMG: Universidade Federal de Minas Gerais; COBEA: Colégio Brasileiro de Experimentação Animal; MG: Minas Gerais; ICB: Instituto de Ciências Biológicas; IFAT: Indirect immunofluorescence antibody test; ELISA: Enzyme linked immunosorbent assay; LSA: *Leishmania* soluble antigen; PCR: Polymerase chain reaction; IHC: Immunohistochemical; HBSS: Hank’s Buffered salt solution; RPMI: Roswell park memorial institute; IEC: Intestinal epithelial cell.

## Competing interests

The authors declare that they have no competing interests.

## Authors’ contributions

MMF, IFGA, AJWP and did all clinical examinations, necropsies, and histology. MMF, VSB, LJP, BD conducted colonoscopy, citometric flow, and morphometrical and statistical analysis. AFC and WLT advisors, revised the histological analysis and the manuscript. All authors read and approved the final manuscript. All authors read and approved the final manuscript.
